# Physiological and Psychological Effects of Short-Term Recreational Football in Adults 60+

**DOI:** 10.3390/ijerph21091194

**Published:** 2024-09-09

**Authors:** Mélanie Boithias, Thi Thao Truc Le, Emma Guillet-Descas, Alain Belli, Mikko Julin, Michael J. Duncan

**Affiliations:** 1 Inter-University Laboratory of Human Movement Biology, Campus Sante, University Jean Monnet Saint-Etienne, 42023 Saint-Etienne, France; alain.belli@univ-st-etienne.fr; 2 Laboratory of Vulnerabilities and Innovation in Sport, UFR STAPS, University Claude Bernard LYON 1, 69100 Villeurbanne, France; le.thithaotruc@gmail.com (T.T.T.L.); emma.guillet@univ-lyon1.fr (E.G.-D.); 3 Research, Development and Innovation Unit, Laurea University of Applied Sciences, 02650 Espoo, Finland; mikko.julin@laurea.fi; 4 Centre for Physical Activity, Sport and Exercise Sciences, Coventry University, Coventry CV1 5FB, UK; aa8396@coventry.ac.uk

**Keywords:** physical activity, aging, modified sport, football, psychology

## Abstract

Recreational football has shown growing evidence that it could be played safely in adults aged 60+ and that it is physically beneficial. Less is known about the psychological aspects, except for the lived experiences of players. The aim of the present study was to analyze both physiological and psychological effects of short-term recreational football. Fifteen participants took part in a six-week training program of recreational football played at a walking pace with two sessions of 1 h and 30 m per week. Physical fitness was assessed before and after the training period and psychological questionnaires were given at the same time. Body mass and body mass index were significantly decreased, but no other significant effects were found on physical fitness. Participants experienced less frustration related to psychological needs (autonomy and competence). Six weeks were too short to observe significant physical improvements while psychological benefits were already experienced. In this short period, psychological aspects seem predominant. These effects may encourage to long-term adhesion. The activity has the potential to keep adults 60+ exercising, which is important for maintaining good global health and seeing physical changes later.

## 1. Introduction

Physical activity (PA) is recommended across the lifespan to maintain good health [1,2]. PA is also known as an effective and preventive strategy to counteract the detrimental adaptations of the aging process. An active lifestyle and participation in sport are protective factors that play a key role in determining the quality of life and overall well-being in young and older adults [3,4].

The physiological effects of regular PA are widely demonstrated in adults aged 65+ [5]. Long-term aerobic programs can improve body composition with less total and abdominal body fat [6]. They result in greater relative muscle mass in limbs [7], higher bone mineral density [8], and increased capacity to transport and use oxygen [9]. Likewise, participation in resistance training programs in older adults resulted in a significant increase in muscle mass and leg strength [10,11].

While the evidence suggests that physical abilities can be improved with specific and appropriate training programs including both aerobic and resistance PA [12], motivation to engage in and maintain regular physical activity is also necessary to achieve optimal and sustained health outcomes. Older adults are becoming more and more aware of the benefits of physical activity on health. Among the factors that limit older adults’ engagement in physical activity, most are linked to health problems such as the fear of falling. In this age group, there is also less self-confidence about physical capacities, and older adults are worried about the safety of physical activity.

Indeed, with advancing age, the satisfaction of the three fundamental psychological needs (autonomy, competence, relatedness) is negatively impacted. The decrease in physical capacities leads to a decrease in autonomy but also in the feeling of competence, and thus in the motivation to engage in physical activities [13]. In addition, at this period of life, it is common to have family members and friends pass away, and the feeling of relatedness is strongly disrupted.

The self-determination theory (SDT), a theory of motivation, postulates that the satisfaction of the three basic psychological needs (autonomy, competence, and affiliation) determines the engagement and quality of life [14]. SDT predicts self-regulated behavior on a continuum on which motivational regulations are classified into two forms of motivation. Autonomous motivation refers to the engagement in behaviors that are consistent with intrinsic goals. This form of motivation is reported as a lever for engagement and maintenance of physical activity, a source of aging well and well-being [13]. On the contrary, in controlled regulations, participation in activities needs external pressures (e.g., punishment, rewards) to persist.

Considering the SDT, to engage older adults in physical activity, training interventions need to act on the three basic psychological needs. A previous study showed that increasing or maintaining older adults’ sense of autonomy and competence produces favorable effects on their well-being as well as their physical and mental health [15]. Numerous studies have shown the positive effect of achieving the basic psychological needs through physical activity and autonomous (vs. controlled) motivation to engage in physical activity on well-being [16].

There remains a gap in investigating the beneficial effects of group interventions in older adults [17]. Most of the training programs offered to older adults targeting physical and functional capacities are individual, with participants targeting their own personal goal; thus, the social aspect is not taken into consideration. In team sports such as football, participants in the team experiment in social connections to achieve a common goal. A recent editorial focused on the beneficial effects of team sports on fitness, health, motivation, and social life, with some studies suggesting that in general, “team sports offer a social and motivational way to improve fitness and health” [18].

Football is a very well-known sport worldwide and different ways of playing have emerged to suit older adults’ physical capacities. Specific rules have been settled to make them safe and tolerable for older adults. One form of football is “walking football”, which differs from recreational football (RF) in terms of intensity. A walking pace is settled and it can easily suit seniors. On the contrary, in RF, slight running is permitted if tolerated by the players. RF is described as a combination of high intensity interval training, endurance training, and strength training with broad-ranging effects on health (cardiovascular, metabolic, and musculoskeletal) [19,20,21]. Recreational football (RF) has been shown to have the potential for long-term health promotion in inactive but football-passionate individuals [22]. In the same way, a recent study concluded that football itself was an intrinsic motivator [23].

RF has gained popularity, especially in older adults for whom it has an anti-inflammatory effect and could slow down biological aging [24]. Previous works have demonstrated that RF was effective after 12 months of training (1.7 sessions per week on average) in older men (mean age = 68.2 years old) by improving bone mineral density and by having positive effects on neuromuscular adaptations, muscle strength, and postural balance [25,26]. The shortest RF programs also reported positive effects in older men. One study showed improvements in functional ability and aerobic fitness after 16 weeks (1.6 sessions per week on average) [27]. Twelve weeks of RF (performed twice a week) enhanced functional movement compared to a control group [28]. Another RF training program of 10 weeks (2 sessions per week) improved exercise tolerance as well as performance in functional capacity tests through increased execution velocity [29].

A few studies also investigated players’ lived experiences. As a team sport, RF has been developed to provide social connections which seem important in this age group. As retired persons, older adults are looking for opportunities to socialize. RF is seen as a grateful and fun activity. Walking football players reported positive experiences and appreciated team identity and collaboration [30,31,32,33].

The effects of RF on older adults’ physical health are increasingly discussed in the literature with different training programs durations. Since 2020, the COVID-19 pandemic has limited the training duration for older people. In a lifestyle where everything has to go fast, there is evidence that short, high intensity programs are a time-efficient training method [34]. It is therefore important to evaluate the effects and efficiency of higher intensity exercises on shorter training periods.

Moreover, there still remains a lack of research examining the effects of RF on psychological parameters, motivation, and quality of life.

To the best of our knowledge, there was no previous study that explores the effects of RF on physical capacities and psychological parameters after a 6-week training program. Moreover, the interaction between physiological and psychological parameters measured before and after a recreational football training intervention in older adults has not been investigated yet.

Even a short training program (6 weeks) could improve physical fitness and decrease body mass in this age group through regular participation in exercise. Given that this activity is recreational, it could easily support interpersonal behaviors of autonomy, competence, and relatedness, which, in turn, could positively influence the satisfaction of the basic psychological needs [35]. Then, this could be reflected in motivational profiles towards more autonomous regulations. Finally, as an enjoyable and fun activity, the quality of life could be increased.

## 2. Materials and Methods

### 2.1. Participants

Following institutional ethical approval of the University Hospital of Saint–Etienne, written informed consent was obtained from the 15 participants (12 men, 3 women; 68.28 ± 5.07 years old; 76.33 ± 12.83 kg; 1.73 ± 0.08 m; 25.49 ± 3.00 kg/m^2^) who were recruited. They were all volunteers. No specific instruction was given about their lifestyle (diet, sleep, hydration, physical activity). Prior to recruitment, three participants were involved in competitive sports and twelve participants were involved in recreational sports.

### 2.2. Procedure

Participants were recruited through fliers dropped in local shops, and a press article was published in the local newspaper. The participants’ sports backgrounds did not matter. The participants who had a successful medical check-up started a cohort study (12-week protocol). Initially, measurements were scheduled at the beginning of the protocol (PRE), after 6 weeks, and after 12 weeks. However, due to the lockdown (COVID-19 pandemic), training sessions were stopped after 8 weeks. Therefore, results from PRE to +6 weeks are discussed in the present article and +6 weeks are considered as POST. Before starting this training intervention, participants completed psychometric questionnaires at their practice facility. Anonymity in the answers, the confidentiality of the obtained data, and their exclusive use for research purposes was assured. The questionnaires were either completed in hard copy or on an online platform (SurveyMonkey software, v.2019). Participants completed several questionnaires before the first training session (PRE) and after their last training session (POST). A member of the research team was present to explain the conditions of the different questionnaires and to answer any questions the participants might have.

### 2.3. Physical Fitness

Physical tests assessed their physical level before (PRE) and after (POST) the 6-week protocol.

For each subject, body mass (kg) and height (m) were measured PRE and POST the 6-week period. Body mass index (BMI) was calculated as mass divided by squared height (kg/m^2^).

Physical tests were from the Senior Fitness Test Manual, which provides functional evaluation tools for people over 60 years old [36]. Only tests for lower limbs were considered. The different tests were the 2.5 Up and Go Test (2.5 UGO), the Chair Sit and Reach Test (S&R), and the 6 Minute Walking Test (6MWT). Two trials were performed for each test, except for the 6MWT, and the best performance was kept for analysis.

### 2.4. Questionnaires

The battery of questionnaires assessed their motivation, level of physical/psychological well-being, subjective quality of life, and expectations of the project.

The French version of the Basic Psychological Need Satisfaction and Frustration (BPNSF) questionnaire was used to assess perceived satisfaction or frustration related to psychological needs [37,38]. This questionnaire is composed of 24 items. Six dimensions were evaluated: autonomy satisfaction competence satisfaction, relatedness satisfaction, autonomy frustration, competence frustration, and relatedness frustration. Participants indicated their agreement with each item through a 5-point Likert scale ranging from 1 (totally disagree) to 5 (totally agree). All items showed good internal consistency, measured with Cronbach’s alpha (Table 1).

The Motivation for Physical Activity for Health Purposes Scale was used to assess the motivation to engage in physical activity for health purposes (EMAPS) [39]. This scale includes 18 items divided into 6 dimensions: intrinsic motivation, integrated regulation, identified regulation, introjected regulation, external regulation, and amotivation. Each item is rated on a 7-level scale ranging from 1 (no match at all) to 7 (very strong match). All items showed good internal consistency, measured with Cronbach’s alpha (Table 2).

The World Health Organization Quality of Life questionnaire (WHOQOL-BREF) assesses the subjective quality of life in relation to several life domains [40]. The WHOQOL-BREF was developed as a generic quality of life questionnaire and is therefore not limited to specific domains of use. It consists of 26 items and includes 4 domains: physical health, psychological well-being, social relationships, and environment. The items are answered on a 5-level scale (e.g., “not at all”, “rather not”, “about”, “most of the time”, “completely”).

### 2.5. Intervention

Each session was performed twice a week, at the same time of the day (10.30 a.m.). Throughout the 60 min, participants performed a complete warm-up with joint mobility exercises and stretching exercises (10–15 min). Then they practiced both football drills or football games to work on the technical and physical aspects (15–20 min). They ended the session with small-sided games (from 2- to 6-a-side, 30–35 min).

### 2.6. Data Analysis

All data were analyzed using JASP version 0.19. Data were tested for normal distribution using the Shapiro–Wilk test. Then, data were analyzed with paired samples tests to investigate the effects of PRE to POST. A Student *t*-test was used for the following variables: 2.5 UGO, S&R, SAU, SAF, FAF, FBC, physical health, psychological, environment, and motivational regulations (integrated, identified, introjected). A non-parametric test (Wilcoxon test) helped for the analysis of other variables: body mass, BMI, 6MWT, FAU, SBC, social relationships, intrinsic motivation, external regulations, and amotivation. All correlation analyses were performed using Pearson’s product–moment correlation or Spearman’s rank correlation rho (non-parametric). Correlation analyses were performed between physiological measurements and psychological parameters before (PRE) and after (POST) training. The *p*-value was set to 0.05 for all tests.

## 3. Results

### 3.1. Physical Fitness

The physiological measurements at PRE and POST are presented in Table 3 (mean ± SD). Body mass and BMI significantly decreased (*p* < 0.05) by 1.44% on average after the training intervention. The performance time of the 2.5 UGO test decreased after 6 weeks of training. Flexibility and number of steps during the 6MWT were higher after the training period. These last physical parameters were improved, but they did not change significantly.

### 3.2. Questionnaires

PRE and POST psychological measurements are presented in Table 4, Table 5 and Table 6 (mean ± SD). Participants experienced significantly less autonomy and competence/need frustration after the training intervention (*p* < 0.05) (Table 4). Autonomy and competence/need satisfaction increased non-significantly (Table 4). Autonomous regulations (identified, integrated, and intrinsic regulations) scores were higher after training, and two controlled regulations (external and amotivation) were lower after 6 weeks of RF. However, all these changes were non-significant (Table 5). Quality of life variables did not change significantly after training (Table 6).

### 3.3. Correlation between Physical Parameters and Psychological Variables

**Figure 1:**
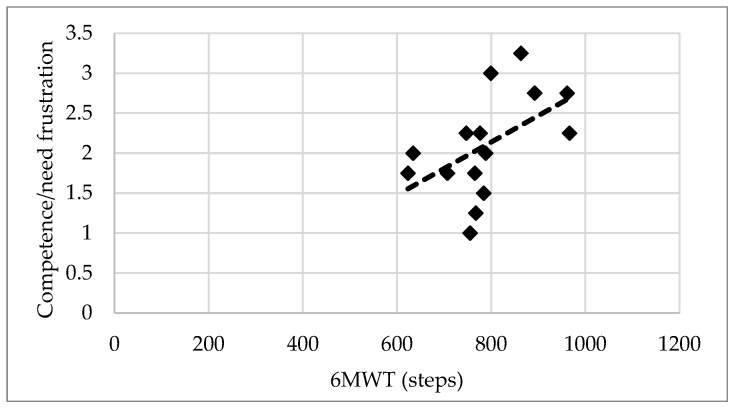
Correlation between competence/need frustration and 6 min walking test (6MWT) performance.

Before training, only a small positive correlation between 6MWT and competence/need frustration (r = 0.516, *p* < 0.05) (Figure 1) and a small negative correlation between S&R and relatedness need satisfaction were observed (r = −0.508, *p* < 0.05).

After training, there were no significant correlations between physiological and psychological parameters.

## 4. Discussion

This study examined the short-term effects of RF on physiological and psychological parameters in older adults aged 60–80 years old. We expected that regular physical activity could improve general physical fitness, and our hypothesis was partially confirmed by the results.

Indeed, our study showed significant decreases in body mass and BMI after 6 weeks of RF. These outcomes find congruence with a longer but still short training program (10 weeks, 2 sessions per week) which decreased body fat percentage in adults from 55 to 70 years old (mean age = 63.5 years old) [29]. A systematic review reported that RF programs shorter than 12 weeks had possibly small beneficial effects on BMI, while the effects on body mass remained unclear [20]. In addition, in adults 65+, the effects of RF on BMI and body mass could possibly be small to largely beneficial [20]. In the current study, the combination of a short training program and an older population has led to significant results for body mass and BMI.

Such positive outcomes have already been observed in specific groups to prevent and control type 2 diabetes by improving body composition with reduced body mass and body fat mass [41]. RF is also effective in young overweight adults to enhance body composition through 16 weeks of training [42]. Our results bring new insights into the possible effects of RF training on body composition in healthy active older adults.

However, other physical parameters seemed to be improved, but the changes were not significant. We could assume that 6 weeks was not enough to significantly impact general physical fitness. However, it must be noticed that participants were all very physically active. According to baselines from Rikli and Jones, they were all above average for their age [36]. Thus, the magnitude of the improvements after training is lower. Still, it remains important to consider the effects on an active population since they could be even larger in a population that is not as fit at the beginning of the program.

Given the recreational and collective nature of the training program, psychological evolutions were expected.

This RF intervention did not significantly influence the basic needs satisfaction, but it significantly decreased the frustration of competence and autonomy (Table 4). Need satisfaction and need frustration, while being assessed in the same questionnaire, seem to be two different constructs because of their different roles [43,44,45]. For instance, previous studies showed that ill-being is related to the presence of need frustration rather than the absence of need satisfaction [38,45,46]. Thus, in our study, the decrease in need frustration could prevent or delay the manifestation of ill-being. Especially in this age group, after retirement, it may be difficult to stimulate the need of competence, as the daily environment may not be as challenging as in the previous working life [47]. In addition, the participation in team sports organized as small-sided games influences the feeling of competence, allowing older adults to develop new skills and creates a challenge, a goal to reach even in a non-competitive activity [48]. Further investigations are needed to understand how these changes can affect competence/need frustration, but RF has the potential to be this new challenging environment.

Considering SDT, the environment and the interpersonal behaviors are important to impact motivation and behaviors [35]. For instance, competence support is needed with structure and feedback provision [49]. Each interpersonal support fosters the satisfaction of its own need but it is also expected that they influence other needs [50]. As well as impacting competence/need, the structure (competence support) settled in RF training can encourage one’s choices, thus improving autonomy. In addition, playing recreationally, football provides freedom in the way that players develop their own skills without external reinforcements; the emphasis is put on options and choices during the exercises. Players do not feel any sense of pressure since the final result does not really matter. These settings described autonomy support that, in turn, foster autonomy need. Considering this environment, it is surprising that autonomy and competence/need satisfaction were improved but non-significantly.

Moreover, no significant effects were found on relatedness. In the current study, players might not have been sensitive to the social aspect because they already experimented it in another way. Since they are active, they could be used to connect to others in their daily life as reported in their low frustration scores and high satisfaction scores. Finally, engaging in team sports might not be enough to develop relatedness. Interpersonal behaviors are also needed to impact the need of relatedness such as, for instance, involvement or encouraging teamwork and collaboration [35,51].

Regarding motivation, in the current study, no significant effects were found on any motivational regulations. It must be noticed that our active older population had at baseline high scores of autonomous regulations and low scores of controlled regulations. These scores were, respectively, increased and decreased, so it could be assumed that a longer training period, as initially scheduled, would have reported significant results. Thus, this assumption is in line with current literature. Team sports organized as small-sided games are more motivating with more intrinsic motives of fun and enjoyment than in individual activities or resistance training [18,48].

Quality of life was improved, but the changes were not significant after the 6-week RF training program. These results could be explained by a lack of item-sensitivity. The questionnaire was about global quality of life and questions were not specific to a training intervention.

Another aim was to investigate the link between physiological and psychological parameters before and after training. Only endurance was correlated to the feeling of competence in older adults before taking part in walking football sessions.

Endurance seems to be an important fitness parameter in older adults since it lets them feel competent even before the training intervention. Indeed, VO_2_max, as a measurement of endurance, determines the ability to produce long efforts [52]. It is really impacted with aging since it decreased by 6–8% per decade, regardless of the initial level of VO_2_max [53]. This decrease could lead to a loss of independence. Thus, it is important to develop endurance, or at least maintain it in older adults. Flexibility was also slightly and significantly correlated to the need of relatedness, but it does not find any scientific and rational explanation in the literature.

Some limitations of the present study should be addressed. Two of them have already been explained. First, the initial program could not come to its end because of lockdown. Then, the participants that volunteered for this training program were highly active for their age and so their physical fitness was already good at baseline, before starting the protocol. This parameter could have been counterbalanced by the fact that none of them knew the activity before, and only some played football in their younger years. Given that they were nearly all beginners, a focus had to be made on the rules during the first sessions; thus, physical demands could have been impacted.

Moreover, the intensity was not monitored during the sessions, which could have helped to adapt the physical demands depending on the players’ current fitness. RPE has already been a useful tool for this purpose [29].

RF training has usually been compared to other forms of training [20]. In the current study, no control group was recruited to explain the effects of the RF training program. The training group was also heterogeneous in terms of gender, with a larger number of men than women. From a physical point of view, RF showed health-related benefits regardless of gender [20]. No research reported the psychological aspects.

With a single assessment of body mass in our study, body composition has not been analyzed in terms of body fat, lean mass, or muscular mass. Assessing the distance covered (m) during the 6MWT could be more accurate.

## 5. Conclusions

In summary, 6 weeks of recreational football on an active older population elicited psychological benefits; however, no significant effects on physical fitness except on body mass and BMI were observed. This is an important observation as an impact on basic psychological needs after a short period may lead to adherence to exercise programs such as recreational football, which in turn facilitates positive changes in physical fitness in healthy older adults. In particular, the decrease in basic need frustration prevents the manifestation of depressive symptoms and ill-being.

Further studies should focus on a longer period of training still, with steady monitoring of psychological parameters with scheduled questionnaires. Then, correlations of changes in physiological and psychological variables could be analyzed. Groups depending on motivational profile could be settled with more participants to study the physical effects among these profiles. Finally, further investigations could explore the effects of gender on basic needs satisfaction and frustration, motivation, and quality of life in RF. It could provide a deeper understanding of the effects on physical fitness and psychological variables in active older adults and settle appropriate RF training programs.

## Figures and Tables

**Table 1:** Internal consistency of the BPNSF questionnaire.

	Satisfaction			Frustration		
Dimension	Autonomy	Competence	Relatedness	Autonomy	Competence	Relatedness
Pre	0.59	0.90	0.90	0.85	0.51	0.77
Post	0.67	0.80	0.85	0.73	0.36	0.75

**Table 2:** Internal consistency of the EMAPS questionnaire.

	Intrinsic	Integrated	Identified	Introjected	External	Amotivation
Pre	0.93	0.93	0.57	0.59	0.97	0.89
Post	0.65	0.86	0.73	0.75	0.72	0.99

**Table 3:** Physiological measurements before (PRE) and after the 6-week walking football training program (POST).

	PRE	POST
Body mass (kg)	76.33 ± 12.83	75.16 ± 13.05 *
BMI (kg/m^2^)	25.49 ± 3.0	25.09 ± 3.05 *
2.5 UGO (s)	4.67 ± 0.51	4.60 ± 0.43
S&R (m)	0.044 ± 0.12	0.048 ± 0.11
6MWT (steps)	788.47 ± 99.85	767.33 ± 141.90

Notes. * Significantly different from pre (*p* < 0.05). Body mass index (BMI), 2.5 Up and Go Test (2.5 UGO), Sit and Reach Test (S&R), 6 min Walking Test (6MWT).

**Table 4:** Basic psychological needs satisfaction and frustration before (PRE) and after the 6-week walking football training program (POST).

	Satisfaction			Frustration		
	Autonomy	Relatedness	Competence	Autonomy	Relatedness	Competence
PRE	3.60 ± 0.74	4.44 ± 0.52	3.72 ± 0.78	2.48 ± 0.91	1.40 ± 0.61	2.10 ± 0.64
POST	3.91 ± 0.56	4.38 ± 0.52	4.07 ± 0.42	2.00 ± 0.64 *	1.27 ± 0.45	1.73 ± 0.50 *

Notes. * Significantly different from PRE (*p* < 0.05).

**Table 5:** Motivational dimensions before (PRE) and after the 6-week walking football training program (POST).

	Motivation					
	Intrinsic	Integrated	Identified	Introjected	External	Amotivation
PRE	5.68 ± 1.41	5.04 ± 1.99	6.21 ± 0.68	4.87 ± 1.31	1.60 ± 1.25	1.79 ± 1.66
POST	5.98 ± 0.64	5.39 ± 1.29	6.31 ± 0.57	5.42 ± 1.19	1.36 ± 0.90	1.36 ± 1.38

**Table 6:** Quality of life dimensions before (PRE) and after the 6-week walking football training program (POST).

	Physical Health	Psychological	Social Relationships	Environment
PRE	27.07 ± 2.84	23.13 ± 3.62	10.87 ± 2.48	30.47 ± 2.13
POST	27.60 ± 2.90	23.53 ± 3.07	11.33 ± 1.23	32.33 ± 3.75

## Data Availability

Data are unavailable due to privacy.
